# Supplementary material for: Muscle niche-driven Insulin-Notch-Myc cascade reactivates dormant Adult Muscle Precursors in Drosophila
Source: eLife. 2015 Dec 9;4:e08497. doi: 10.7554/eLife.08497 (PMC4749548; doi:10.7554/eLife.08497)
Supplement: Figure 4—source data 1. — For each genotype, the average number of cells ± standard error mean is shown. Sample size (n) is indicated in brackets (Rebay et al., 1993). DOI: http://dx.doi.org/10.7554/eLife.08497.015 [file elife-08497-fig4-data1.docx]

**Figure 4-figure supplement 2.**

|  | Dorsal | Lateral | Ventral |
| --- | --- | --- | --- |
| *M6-Gal4* | 14,2 ± 1,7 (28) | 16,2± 1,3 (28) | 8,8 ± 1,3 (28) |
| *M6>InRCA* | 21,2± 2,8 (28) | 26,3 ± 2,5 (27) | 8,8± 0,9 (30) |
| *M6>PTEN* | 7 ± 2,2 (29) | 7,6 ± 1,9 (30) | 6± 1,4 (31) |
| *M6>RHEB* | 20,4± 2,2 (26) | 26,2 ± 3,9 (27) | 9,1 ± 1,0 (25) |
| *M6>TSC1,TSC2* | 6 ± 1,5 (26) | 7,8 ± 1,1 (29) | 4,8 ± 1,0 (26) |
| *M6>NICD* | 52,5± 6,2 (28) | 46,3 ± 5,1 (28) | 8,3 ± 1,1 (28) |
| *M6>NotchRNAi* | 5,8± 2,2 (28) | 9,2 ± 2,8 (28) | 5,5 ± 1,4 (28) |
| *M6>dMyc* | 25,9± 2,8 (28) | 27,5 ± 3,5 (29) | 9,8 ± 0,8 (29) |
| *M6>dMycRNAi* | 2,6± 0,4 (28) | 2,9± 0,6 (31) | 2 ± 0,2 (24) |
| *M6>NumbRNAi* | 15,86± 0,28 (29) | 16,48± 0,25 (29) | 8,68± 0,15 (29) |
