# Supplementary material for: Muscle niche-driven Insulin-Notch-Myc cascade reactivates dormant Adult Muscle Precursors in Drosophila
Source: eLife. 2015 Dec 9;4:e08497. doi: 10.7554/eLife.08497 (PMC4749548; doi:10.7554/eLife.08497)
Supplement: Figure 5—source data 1. — For each genotype, the average number of cells ± standard error mean is shown. Sample size (n) is indicated in brackets. Below a table with mean signal intensity measurements for Myc and NICD in lateral AMP cluster from the genotypes shown in Figure 5B–D'',J,K. For each genotype, the average signal intensity value ± standard error mean is shown. Sample size (n) is indicated in brackets. DOI: http://dx.doi.org/10.7554/eLife.08497.019 [file elife-08497-fig5-data1.docx]

**Figure 5-figure supplement 2.**

AMPs number

|  | Dorsal | Lateral | Ventral |
| --- | --- | --- | --- |
| *M6-Gal4* | 14,2 ± 1,7 (28) | 16,2± 1,3 (28) | 8,8 ± 1,3 (28) |
| *M6>InRCA; lacZ* | 19,1± 2,2 (27) | 26,9± 2,7 (29) | 7,96 ± 0,67 (30) |
| *M6>InRCA; NotchRNAi* | 10,7± 1,9 (26) | 12,9 ± 2,4 (26) | 7,4± 1,1 (25) |
| *M6>InRCA; dMycRNAi* | 3,3± 0,5 (28) | 4,4 ± 0,85 (32) | 3,5± 1,0 (29) |
| *M6>NICD; lacZ* | 39,5 ± 3,6 (31) | 46 ± 5,1 (31) | 9,9 ± 0,75 (31) |
| *M6>NICD; dMycRNAi* | 7,8± 0,71 (29) | 6,9± 1,2 (29) | 5 ± 0,83 (29) |
|  |  |  |  |

Signal intensity

|  | Myc | NICD | | |  |
| --- | --- | --- | --- | --- | --- |
| *M6-Gal4* | 64,6 ± 9,8 (12) | | | 46,8± 12,6 (12) | |
| *M6>InRCA* | 102,6± 14,2 (14) | | | 101,9± 16,1 (14) | |
| *M6>PTEN* | 16,2± 4,6 (14) | | | 24,7 ± 8,4 (14) | |
| *M6>NICD* | 95,1± 8,4 (15) | | - | |  |
| *M6>NotchRNAi* | 9,3 ± 1,2 (14) | | - | |  |
