# Supplementary material for: Muscle niche-driven Insulin-Notch-Myc cascade reactivates dormant Adult Muscle Precursors in Drosophila
Source: eLife. 2015 Dec 9;4:e08497. doi: 10.7554/eLife.08497 (PMC4749548; doi:10.7554/eLife.08497)
Supplement: Figure 6—source data 1. — For each genotype, the average number of cells ± standard error mean is shown. Sample size (n) is indicated in brackets. Below, a table with mean Deltex signal intensity measurements in lateral AMP cluster from the genotypes shown in Figure 6A–C. For each genotype, the average signal intensity value ± standard error mean is shown. Sample size (n) is indicated in brackets. DOI: http://dx.doi.org/10.7554/eLife.08497.022 [file elife-08497-fig6-data1.docx]

**Figure 6-figure supplement 3.**

AMPs number

|  | Lateral |
| --- | --- |
| *M6-Gal4* | 16,2± 1,3 (30) |
| *M6>Dx* | 21,16± 0,56 (30) |
| *M6>Su(Dx)RNAi* | 23± 0,54 (30) |
| *M6>ShrubRNAi* | 29,06 ± 0,6 (30) |
| *M6>InRCA; lacZ* | 26,9± 2,7 (30) |
| *M6> InRCA; Dx* | 31,63± 0,51 (30) |
| *M6> PTEN* | 7,6 ± 1,9 (30) |
| *M6> PTEN; Dx* | 15,4± 0,44 (30) |
|  |  |
| *Elav>DeltaRNAi* | 16,4 ± 0,26 (29) |
| *Elav>SerrateRNAi* | 16,86± 0,23 (29) |
| *Mef>DeltaRNAi* | 16,35± 0,19 (30) |
| *Mef>SerrateRNAi* | 17,19 ± 0,03 (30) |
| *M6> NotchDN* | 15,45± 0,24 (30) |
| *Elav>DeltaRNAi* | 16,4 ± 0,26 (29) |
| *Mef-Gal4* | 16,1 ± 0,9 (30) |
| *Elav-Gal4* | 16,2± 0,33 (30) |
|  |  |
| *M6-Gal4* | 16,2± 1,3 (30) |
| *M6>Su(Dx)* | 23,7± 0,69 (30) |
| *M6>Krz* | 24,6 ± 0,71 (30) |
| *M6>INRCA;Krz* | 30,96 ± 0,8 (30) |
| *M6>DxRNAi* | 26,3 ± 0,48 (30) |
| *M6> KrzRNAi* | 24,2± 0,62 (30) |
| *M6> Dx;Krz* | 17,6 ± 0,41 (30) |
|  |  |
|  |  |
|  |  |

Signal intensity

|  | Deltex |
| --- | --- |
| *M6>lacZ* | 12,78 ± 0,53 (16) |
| *M6>InRCA* | 29,41± 2,04 (11) |
