# Supplementary material for: Muscle niche-driven Insulin-Notch-Myc cascade reactivates dormant Adult Muscle Precursors in Drosophila
Source: eLife. 2015 Dec 9;4:e08497. doi: 10.7554/eLife.08497 (PMC4749548; doi:10.7554/eLife.08497)
Supplement: Figure 7—source data 1. — For each genotype, the average number of cells ± standard error mean is shown. Sample size (n) is indicated in brackets. DOI: http://dx.doi.org/10.7554/eLife.08497.026 [file elife-08497-fig7-data1.docx]

**Figure 7-figure supplement 2.**

|  | Dorsal | Lateral | Ventral |
| --- | --- | --- | --- |
| *Df-dIlp6* | 7,7± 0,21 (30) | 7,8± 0,2 (30) | 7,5 ± 0,17 (30) |
| *Df-dIlp2* | 12,4± 0,33 (30) | 13,8 ± 0,29 (30) | 8,2± 0,15 (30) |
| *Df-dIlp5* | 14± 0,24 (30) | 16,4 ± 0,25 (30) | 9,4± 0,17 (30) |
| *Wt (Canton S)* | 14,5 ± 0,7 (30) | 15,9± 0,55 (30) | 8,3 ± 0,3 (30) |
| *Mef-Gal4* | 14,8 ± 0,9 (30) | 16,1 ± 0,9 (30) | 9,1 ± 0,75 (30) |
| *Elav-Gal4* | 15,1± 0,7 (30) | 16,2± 0,33 (30) | 7,4± 0,83 (30) |
| *Repo-Gal4* | 14,9± 0,62 (30) | 16,3± 0,4 (30) | 8,3 ± 0,53 (30) |
| *Mef>ShiDN* | 7± 0,21 (25) | 7,5± 0,22 (24) | 4,7 ± 0,14 (26) |
| *Elav>ShiDN* | 15,7± 0,31 (24) | 18± 0,37 (23) | 8,9 ± 0,18 (25) |
| *Repo>ShiDN* | 17,5± 0,27 (24) | 18,1± 0,31 (24) | 9± 0,2 (22) |
| *Mef>dIlp6RNAi* | 9,2± 0,23 (26) | 9,5± 0,22 (24) | 7,6 ± 0,18 (22) |
| *Repo>dIlp6RNAi* | 14,4± 0,36 (30) | 17,1± 0,27 (30) | 9 ± 0,27 (30) |
| *Mef>dIlp6* | 30,5± 0,63 (26) | 26,4± 0,78 (27) | 10,1 ± 0,15 (25) |
| *M6-Gal4* | - | 16,2± 1,3 (30) | - |
| *M6>DAAMRNAi* | - | 10,6± 0,61 (30) | - |
